# Increasing the Biological Stability Profile of a New Chemical Entity, UPEI-104, and Potential Use as a Neuroprotectant Against Reperfusion-Injury

**DOI:** 10.3390/brainsci5020130

**Published:** 2015-04-21

**Authors:** Tarek M. Saleh, Barry J. Connell, Inan Kucukkaya, Alaa S. Abd-El-Aziz

**Affiliations:** 1 Department of Biomedical Sciences, Atlantic Veterinary College, Charlottetown, PE C1A 4P3, Canada; E-Mail: Connell@upei.ca; 2 Department of Chemistry, University of Prince Edward Island, Charlottetown, PE C1A 4P3, Canada; E-Mails: ikucukkaya@upei.ca (I.K.); abdelaziz@upei.ca (A.S.A.)

**Keywords:** stroke, ischemia/reperfusion, middle cerebral artery occlusion, antioxidant therapy, neuroprotection, bio cleavage-resistance

## Abstract

Previous work in our laboratory demonstrated the utility of synthetic combinations of two naturally occurring, biologically active compounds. In particular, we combined two known anti-oxidant compounds, lipoic acid and apocynin, covalently linked via an ester bond (named UPEI-100). In an animal model of ischemia-reperfusion injury (tMCAO), UPEI-100 was shown to produce equivalent neuroprotection compared to each parent compound, but at a 100-fold lower dose. However, it was determined that UPEI-100 was undetectable in any tissue samples almost immediately following intravenous injection. Therefore, the present investigation was done to determine if biological stability of UPEI-100 could be improved by replacing the ester bond with a more bio cleavage-resistant bond, an ether bond (named UPEI-104). We then compared the stability of UPEI-104 to the original parent compound UPEI-100 in human plasma as well as liver microsomes. Our results demonstrated that both UPEI-100 and UPEI-104 could be detected in human plasma for over 120 min; however, only UPEI-104 was detectable for an average of 7 min following incubation with human liver microsomes. This increased stability did not affect the biological activity of UPEI-104 as measured using our tMCAO model. Our results suggest that combining compounds using an ether bond can improve stability while maintaining biological activity.

## 1. Introduction

Reactive oxygen species (ROS) play an important role in the development of neurovascular diseases, including stroke, dementia, multiple sclerosis, and Parkinson’s disease. This is due, in large part, to excess production of oxidants, decreased nitric oxide (NO) bioavailability, and decreased antioxidant capacity in the vasculature of the central nervous system [1,2,3,4]. One of the major sources for vascular ROS is a family of nonphagocytic nicotinamide adenine dinucleotide phosphate (NADPH) oxidases (Nox), including the prototypic Nox2 homolog-based NADPH oxidase [5].

There is considerable interest in the anti-oxidative and anti-inflammatory effects of phenolic compounds from different botanical sources. The evaluation of neuroprotective effects of phenolic compounds is gaining considerable interest as therapeutic agents [6,7]. Some examples include resveratrol from grape and red wine, curcumin from turmeric, apocynin, and epi-gallocatechin from green tea [8,9]. Apocynin (4-hydroxy-3-methoxyacetophenone) is a major active ingredient from the rhizomes of *Picorrhiza kurroa*, a botanical plant used as an herbal medicine for potential treatment of a number of inflammatory diseases [10]. Recently, apocynin is regarded as a specific inhibitor for NADPH oxidase in cell and animal models. There have been reports that have demonstrated an attenuation of infarct volume when apocynin was administered during an occlusion of the middle cerebral artery (MCAO), but these benefits were lost when administration of apocynin was delayed following the onset of reperfusion [10,11,12,13,14]. However, one laboratory demonstrated that administration of apocynin in gerbils 5 min following 5 min of global ischemia, decreased neuronal degeneration and delayed neuronal death and microglial activation when assessed four days later [15]. It is not known whether these apocynin-induced beneficial effects translated to a decrease of infarct size, as it was not measured in that study.

α-Lipoic acid (LA) is a naturally occurring eight-carbon fatty acid that is synthesized by plants and animals, including humans. LA has been reported to lower serum triglycerides, increase glucose uptake by cells, stimulate neurological function, decrease liver toxicity, increase levels of glutathione and ascorbic acid and decrease the expression of inflammatory molecules [16,17,18]. LA has also been shown to have a neuroprotective effect [19,20]. LA has a very short half-life in the bloodstream as it undergoes rapid metabolism in the liver [21]. Dosing 3–4 times daily is necessary to accomplish reasonable blood levels. This rather limited bioavailability of LA could be extended by suitably incorporating chemical groups to attenuate the metabolic process in the liver [22,23]. Previous studies have demonstrated that administration of the most effective dose of LA (5 mg/kg) did not result in significant neuroprotection when administered just prior to the beginning of reperfusion, and only produced neuroprotection when administered prior to both occlusion and reperfusion [19].

Recently, combinations of LA with several bioactive compounds have gained interest as a therapeutic strategy for many chronic diseases. It was recently demonstrated in humans that the combination of LA with the angiotensin receptor blocker irbesartan markedly reduced proinflammatory soluble IL-6 and VCAM-1 levels and improved vascular endothelial function [24]. Covalent linkage of LA with ibuprofen has been demonstrated to be neuroprotective in rodent models of Alzheimer’s disease in which administration of the co-drug decreased the oxidative damage due to the infusion of Aβ (1–40) [25]. In addition, a co-drug produced by chemically linking LA with l-Dopa, or dopamine, decreased neuronal oxidative damage associated with the administration of l-Dopa or dopamine alone [26].

Apocynin-lipoic acid conjugates have recently been described [27]. This compound, named UPEI-100, is an apocynin-lipoic acid conjugate with an ester linkage between the apocynin and lipoic acid components of the co-drug. This ester linked compound proved to be very unstable in that it could not be detected in the central nervous system immediately following intravenous injection. Therefore, the present study was designed to synthesize a covalent conjugate between LA and apocynin comprising a bio cleavage-resistant conjugate linkage (ether linkage herein named “UPEI-104”). We then compared the stability profile of this compound with the previous ester bonded compound, UPEI-100 in both human plasma and human liver microsomes. Finally, the efficacy of this new compound, UPEI-104, in our *in vivo* model of ischemia-reperfusion injury was tested.

## 2. Experimental Section

### 2.1. Surgical Procedures

All surgical procedures described below were approved by the University of Prince Edward Island Animal Care Committee and were in compliance with the requirements of the Canadian Council on Animal Care. Our surgical protocol has been described previously [28]. Briefly, rats were anaesthetized with sodium thiobutabarbital (Inactin, Sigma Aldrich, St. Louis, MO, USA; 100 mg/kg, intraperitoneal; i.p.) and kept warm on a heating pad maintained at 37 ± 1 °C. A venous cannula was inserted into the right femoral vein to allow for all drug injections and supplemental anaesthesia, if required. Rats were then transferred to a stereotaxic frame to allow for surgical access to the right middle cerebral artery (MCA). As detailed in our previous publication [28], we used a 3-point occlusion of the MCA by inserting suture under the vessel and applying pressure, by gently lifting the sutures, to occlude flow by 100% (as confirmed by laser Doppler flowmetry). The period of occlusion lasted for 30 min, followed by a 5.5 h period of reperfusion, which was accomplished by removal of these sutures (transient ischemia model, tMCAO).

Following the 5.5 h of reperfusion, rats were sacrificed and brains were removed and sliced into a series of 1 mm coronal sections through the area of infarct using a rat brain matrix (Harvard Apparatus; Holliston, MA, USA). Sections were subsequently immersed in a 2% solution of TTC for 5 min followed by immersion overnight in 10% formalin fixative. Sections were then scanned (both sides) the next day, and infarct area was measured using imaging software (Image J; Scion Corporation, Frederick, MD, USA). The average area for each section was multiplied by section thickness (1 mm) to provide an infarct volume. The sum of volume measurements for all sections from each brain provided a total infarct volume for each animal. Data was analyzed by an investigator blinded to the treatment groups.

### 2.2. Dose-Response Curve for UPEI-104

A dose-response curve for UPEI-104 was generated by preparing a stock solution, which was then used to produce all concentrations used (0.001–0.1 mg/kg; *n* = 7 or 8 per group). Serial dilutions were made using 25% ethanol. Therefore we used 25% ethanol as our vehicle control. All drug or vehicle injections were made via the intravenous catheter 30 min prior to occlusion of the MCA (*n* = 7–8/dose group).

### 2.3. Reagents and Animals

Lipoic acid, apocynin, 2,3,5-triphenol tetrazolium chloride (TTC), and all reagents required for the synthesis of UPEI-104 (detailed below) were purchased from Sigma Aldrich (St. Louis, MO, USA). Male Sprague-Dawley rats (250–350 g) were purchased from Charles River Laboratories (Montreal, PQ, Canada).

### 2.4. UPEI-100 and -104 Synthesis Method

**Figure 1:**
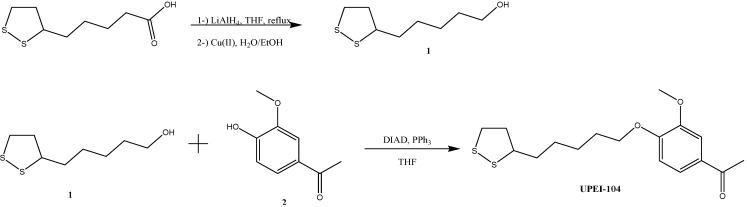

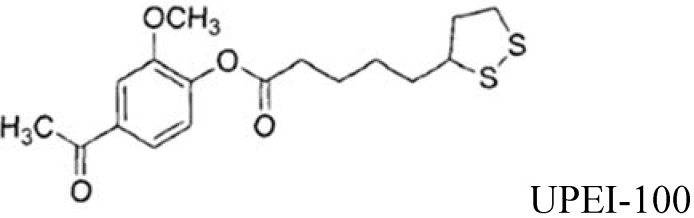
Summary of the synthetic method (including intermediate compounds; **1** and **2**) in the synthesis of the ether-linked apocynin-lipoic acid conjugate (UPEI-104). Insert shows UPEI-100 for comparison. A full synthetic description for UPEI-100 has been previously described [27].

UPEI-100 was synthesized as previously described [27] and is shown below for comparison (Figure 1; insert). For the synthesis of UPEI-104, lipoic alcohol was prepared by reduction of lipoic acid with LiAlH_4_ followed by oxidation using Cu(II) according to Algar and Krull [29]. Purity was confirmed by proton nuclear magnetic resonance spectroscopy (^1^H NMR). Lipoic alcohol (Figure 1; compound **1**; 1 mmol), triphenylphosphine (PPh3; 1.3 mmol) and apocynin (Figure 1; compound **2**; 1 mmol) in tetrahydrofuran (THF; 20 mL) were gently stirred for 5 min at 0 °C under inert atmosphere. Diisopropyl azodicarboxylate (DIAD; 1.3 mmol) was added dropwise and the reaction mixtures were stirred at room temperature overnight. The solvent was removed by evaporation and crude compound was purified by collecting the first fraction on a silica column chromatography (R_f_:0.76, Eluent, Hexanes:Ethylacetate 1:1):yellow oil, Yield; 73%. ^1^H NMR (300 MHz, CDCl_3_) δ 7.67–7.50 (m, 2H), 7.13 (d, *J* = 8.1 Hz, 1H), 3.90 (s, 3H), 3.60 (dd, *J* = 13.6, 7.1 Hz, 1H), 3.27–3.08 (m, 2H), 2.65 (d, *J* = 7.3 Hz, 2H), 2.61 (s, 3H), 2.50 (dd, *J* = 12.4, 6.0 Hz, 1H), 1.94 (dd, *J* = 12.8, 6.8 Hz, 1H), 1.88–1.66 (m, 6H), 1.66–1.49 (m, 2H).

### 2.5. Stability in Human Plasma

Samples of UPEI-100 and UPEI-104 were sent to Cerep Inc., (Redmond, WA, USA), a preclinical contract research organization, for stability testing in human plasma and human liver microsomes. The metabolic profile of both UPEI-100 and UPEI-104 were first evaluated in human plasma. Reference compounds (propantheline, propoxycaine and verapamil) were also tested to provide positive control data for these assays. Incubation was performed at 37 °C, for times between 0 and 120 min as indicated. The detection method was HPLC-MS/MS. At the end of incubation at each of the time points, acetonitrile was added to the incubation mixture followed by centrifugation. Samples were analyzed by HPLC-MS/MS and peak areas were recorded for each analyte. The area of precursor compound remaining after each of the time points relative to the amount remaining at time zero, expressed as percent, was calculated. Subsequently, the half-life (T_1/2_) was estimated from the slope of the initial linear range of the logarithmic curve of compound remaining (%) *vs.* time, assuming first order kinetics. Further experimental detail may be found in [30].

### 2.6. Stability in Human Liver Microsomes

Next, the metabolic profile and intrinsic clearance of UPEI-100 and UPEI-104 were tested by Cerep Inc., (as above) using human liver microsomes. Reference compounds (Imipramine, propranolol, terfenadine and verapamil) were also tested to provide positive control data for this assay. The details of the assay used to compare the metabolic stability in human liver microsomes (0.1 mg/mL) of UPEI-104 and UPEI-100 are provided in [31]. Briefly, incubation was performed at 37 °C for times between 0 and 60 min as indicated. The detection method was HPLC-MS/MS. Intrinsic clearance from microsomes (S9, cryopreserved hepatocytes, recombinant CYP, recombinant UGT) was assayed. Metabolic stability, expressed as percent of the parent compound remaining, was calculated by comparing the peak area of the compound at the time point relative to that at time 0. The half-life (T_1/2_) was estimated from the slope of the initial linear range of the logarithmic curve of compound remaining (%) *vs.* time, assuming the first-order kinetics. The apparent intrinsic clearance (CLint, in μL/min/pmol, μL/min/mg or μL/min/Mcell) was calculated according to the following formula: ${\text{CL}}_{\text{int}}=\frac{0.693}{{\text{T}}_{\text{1/2 }}(\text{mg protein}/\text{μL or million cells}/\text{μL or pmol CYP isoyme}/\text{μL })}$

### 2.7. HPLC-MS Optimization

A solution of each test compound (1000 ng/mL or 10 μM) was prepared as described above and infused into the TSQ Quantum source via syringe pump at a constant rate. Full scan MS analysis was conducted and total ion current chromatograms and corresponding mass spectra were generated for each test compound in both positive and negative ionization modes. The precursor ions for MS/MS were selected from either the positive or the negative mass spectrum, as a function of the respective ion abundance. In addition, product ion MS/MS analysis was performed in order to determine the appropriate selected fragmentation reaction for use in quantitative analysis. The final reaction monitoring parameters were chosen to maximize the ability to quantify the test compound when present within a complex mixture of components. Following identification of the specific SRM transition to be used for each test compound, the detection parameters were optimized using the automated protocol in the TSQ Quantum Compound Optimization Workspace software package (Thermo Scientific, MA, USA). Finally, the chromatographic conditions to be used for LC-MS analysis were identified by injection and separation of the analyte on a suitable LC column and adjustment of the gradient conditions as necessary. Further experimental detail may be found in [32]. All samples were analyzed on TSQ Quantum with selected reaction monitoring, positive ion mode, capillary temperature 325 °C, capillary voltage 4 kV. A parent ion 355.4 m/z [M + H]^+^ was a low intensity peak. The retention time of this peak was 1.74 min (collision offset of −10 V; product ion 189.3 m/z). 

### 2.8. HPLC Conditions:

Mobile Phase A: 13.3 mM ammonium formate/6.7 mM formic acid in water. Mobile Phase B: 6 mM ammonium formate/3 mM formic acid in water/acetonitryl (ACN; 1/9, v/v). Column: Hypersil GOLD DASH HTS, 2.1 × 20 mm, 3 micron (Thermo Scientific, MA, USA; see Table 1 for details).

**Table 1:** Gradient Program.

Time, min	% B	Flow rate, mL/min
0	0	0.5
1.5	100	0.5
2.5	100	0.5
2.6	0	0.5
3.0	0	0.5

### 2.9. Statistical Analysis

All data are presented as mean ± S.E.M. Data were analyzed by a one-way ANOVA, followed by a Bonferroni post-hoc test. When comparing 2 groups only, a Student’s *t*-test was used. In all cases, *p* values ≤ 0.05 were considered statistically significant.

## 3. Results

### 3.1. Comparison of Stability of UPEI-100 vs. UPEI-104 in Human Plasma

The results demonstrate that both UPEI-100 and UPEI-104 have a half-life in human plasma under the experimental conditions of greater than 120 min (Table 2).

**Table 2:** Metabolic stability of UPEI-104 and UPEI-100 in human plasma.

**Compound**	**Test Concentration**		**Incubation Time(minutes)**	**% Compound Remaining**					**Half-Life (minute)**			
				**1st**		**2nd**		**Mean**	**1st**	**2nd**		**Mean**
UPEI-100	1.0E−06 M		0	100.0	100.0		100		>120	>120		>120
UPEI-100	1.0E−06 M		30	92.7	126.6		110		-	-		-
UPEI-100	1.0E−06 M		60	100.7	116.0		108		-	-		-
UPEI-100	1.0E−06 M		90	99.5	111.2		105		-	-		-
UPEI-100	1.0E−06 M		120	109.9	140.1		125		-	-		-
UPEI-104	1.0E−06 M		0	100.0	100.0		100		351.8	351.1		>120
UPEI-104	1.0E−06 M		30	89.3	112.0		101		-	-		-
UPEI-104	1.0E−06 M		60	95.5	98.6		97		-	-		-
UPEI-104	1.0E−06 M		90	82.9	76.4		80		-	-		-
UPEI-104	1.0E−06 M		120	77.2	90.1		84		-	-		-
**Reference Compound**		**Test** **Concentration**		**Half-Life(minute)**								
				**1st**			**2nd**			**Mean**		
**Half-life (plasma, human)**												
Propantheline		1.0E−06 M		7.2			7.2				7		
Propoxycaine		1.0E−06 M		<30			<30				<30		
Verapamil		1.0E−06 M		1410.1			2004.7				>120		

### 3.2. Intrinsic Clearance Metabolic Profile in Human Liver Microsomes

The results demonstrate that UPEI-104 has a half-life in human liver microsomes under the experimental conditions of approximately 7 min. UPEI-100, on the other hand, was not detected in the assay at any time point (Table 3).

**Table 3:** Metabolic stability/intrinsic clearance (*CL*_int_) of UPEI-104 and UPEI-100 in human liver microsomes.

Compound	Test Concentration	Incubation Time(minutes)	% Compound Remaining					Half-Life (minute)			Clint	Flags
			1st		2nd		Mean	1st	2nd	Mean		
**Intrinsic clearance (liver microsomes, human)**												
UPEI-100	1.0E−07 M	0	0.0	0.0		0		-	-	-	-	ND
UPEI-100	1.0E−07 M	15	0.0	0.0		0		-	-	-	-	ND
UPEI-100	1.0E−07 M	30	0.0	0.0		0		-	-	-	-	ND
UPEI-100	1.0E−07 M	45	0.0	0.0		0		-	-	-	-	ND
UPEI-100	1.0E−07 M	60	0.0	0.0		0		-	-	-	-	ND
UPEI-104	1.0E−07 M	0	100.0	100.0		100		7.3	7.1	7	962.7	-
UPEI-104	1.0E−07 M	15	34.6	34.8		35		-	-	-	-	-
UPEI-104	1.0E−07 M	30	8.8	5.8		7		-	-	-	-	-
UPEI-104	1.0E−07 M	45	1.4	1.4		1		-	-	-	-	-
UPEI-104	1.0E−07 M	60	1.1	1.2		1		-	-	-	-	-
**Reference Compound**	**Test Concentration**	**Half-Life (minute)**								**Clint**		
		**1st**		**2nd**			**Mean**					
**Intrinsic clearance (liver microsomes, human)**												
Imipramine	1.0E−07 M	231.4		174.4			>60			<115.5		
Propranolol	1.0E−07 M	264.2		273.1			>60			<115.5		
Terfenadine	1.0E−07 M	6.8		7.7			7			959.1		
Verapamil	1.0E−07 M	22.5		21.6			22			314.3		

Note: Unit of Clint is μL/min/mg for microsomes, S9 and UGT assays; μL/min/pmol for CYP assays; μL/min/Million cells for hepatocyte assays; ND: Not Detected. Test compound was not reliably detected in the assay matrix.

Following optimization of the HPLC protocol to improve UPEI-100 detection, the experiment was repeated and results are shown in Table 4. As can be seen in Table 4, UPEI-100 was only detected for a mean of ~1 min in human liver microsomes.

**Table 4:** Metabolic stability/intrinsic clearance (Cl_int_) of UPEI-100 in human liver microsomes.

Compound		Test Concentration		Incubation Time(minutes)	% Compound Remaining							Half-Life (minute)						Clint		
					1st			2nd		Mean		1st		2nd			Mean			
**Intrinsic clearance (liver microsomes, human)**																				
UPEI-100		1.0E−07 M		0	100.0		100.0		100			1.1	<15			1		6032.4		
UPEI-100		1.0E−07 M		15	0.0		0.0		0			-	-			-		-		
UPEI-100		1.0E−07 M		30	0.0		0.0		0			-	-			-		-		
UPEI-100		1.0E−07 M		45	0.0		0.0		0			-	-			-		-		
UPEI-100		1.0E−07 M		60	0.0		0.0		0			-	-			-		-		
**Reference Compound**		**Test Concentration**			**Half-Life(minute)**													**Clint**
					**1st**					**2nd**				**Mean**				
**Intrinsic clearance (liver microsomes, human)**																		
Imipramine		1.0E−07 M			216.3					135.4				>60				<115.5
Propranolol		1.0E−07 M			132.1					137.3				>60				<115.5
Terfenadine		1.0E−07 M			9.4					9.1				9				748.1
Verapamil		1.0E−07 M			19.7					21.6				21				336.2

Note: Unit of Clint is μL/min/mg for microsomes, S9 and UGT assays; μL/min/pmol for CYP assays; μL/min/Million cells for hepatocyte assays.

### 3.3. Dose-Dependent Effects of UPEI-104 on Infarct Volume Following tMCAO

**Figure 2:**
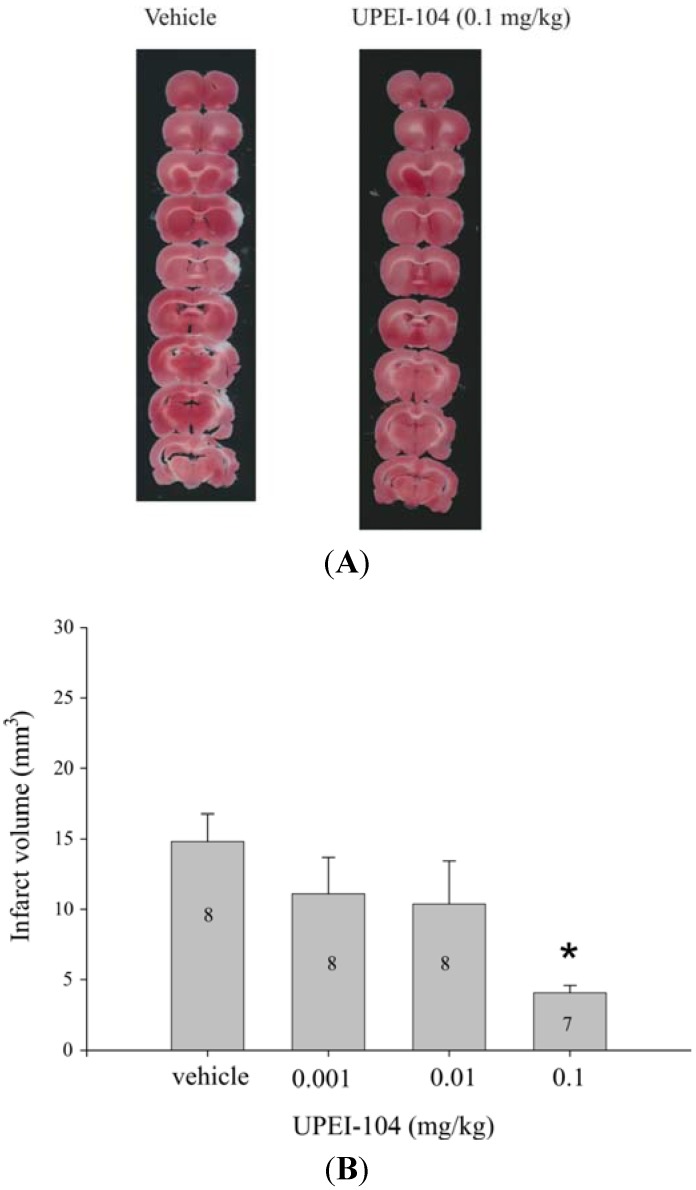
(**A**) Representative photomicrographs of 2,3,5-triphenol tetrazolium chloride (TTC)-stained, 1 mm thick coronal sections through the rat brain. Lack of staining (white regions) in the tissue from both vehicle and UPEI-104-treated rats indicates areas of infarct; (**B**) Bar graph demonstrating the effect of UPEI-104 treatment on infarct volume as a function of dose in Sprague-Dawley rats that were treated with the compound (0.001–0.1 mg/kg) 30 min prior to the middle cerebral artery (MCA) was occluded to induce a stroke. Numbers inside bars indicate the number of animals/dose group. (* Asterisk indicates significantly different from vehicle group; *p* < 0.05; ANOVA).

Pre-treatment with UPEI-104 at 30 min prior to MCA occlusion resulted in a dose-dependent reduction in infarct volume when measured following ischemia/reperfusion. Infarct volume was significantly lower in rats treated with 0.1 mg/kg UPEI-104 (Figure 2). This dose was equal to that previously observed with the parent compound, UPEI-100, when tested using the same *in vivo* model of reperfusion-injury in our laboratory [27].

## 4. Discussion 

Previous work in our laboratory has provided evidence that pre-administration of apocynin and lipoic acid at sub-threshold levels for neuroprotection enhanced the neuroprotective capacity when injected in combination [33]. We then showed that synthetically combining these compounds via an ester bond (UPEI-100), significantly improved their biological activity even further [27]. Unfortunately, we were unable to detect UPEI-100 in tissue samples of the animals injected with the compound, even as early as 1 min following intravenous injection. This suggested that the compound UPEI-100 was not able to remain intact following first pass metabolism though the liver. The present study was carried out to determine if a lipoic acid-apocynin co-drug comprised of lipoic acid and apocynin functional groups bound by a covalent linkage designed to have improved stability *in vivo,* would be capable of maintaining bioactivity.

A covalent linkage between lipoic acid (LA) and apocynin may contain a number of bio cleavage-resistant linkages having a suitable metabolic profile such that the lipoic acid and apocynin components of the conjugate remain linked *in vitro* or *in vivo* for at least a brief duration of time. However, it is unknown if alterations in the nature of these linkages affect biological activity *in vivo*. In our study, we selected an ether bond as the covalent linkage so as to provide lipoic acid-apocynin conjugates having a half-life in human plasma of greater than 120 min, or a half-life in human liver microsomes of greater than or equal to about 7 min, as measured according to assays described herein. We feel that this ether bond may provide a more stable covalent linkage, which is at least partially resistant to bio cleavage and/or degradation *in vitro* and/or *in vivo* as compared to ester linkages (UPEI-100) or disulphide linkages. Our results demonstrate that UPEI-104, the ether bonded compound, had significantly improved stability without compromising bioactivity. The results shown in Figure 2 indicate that UPEI-104, (ether-linked LA-apocynin conjugate), has similar activity at the same dose as that previously observed using the ester linked conjugate (UPEI-100) in terms of reduction in stroke infarct volume [27].

The lack of plasma stability of UPEI-100 is most likely due to the ester bond, as ester bonds are most susceptible to bio cleavage by esterases in the blood. Vertebrate plasma contains three main forms of esterases; cholinesterases, arylesterases and aliesterases [34]. In contrast, ether linkages are significantly less susceptible to enzymatic cleavage as there are no specific enzymes that control this, however, hydrolases are capable of metabolising/cleaving ether bonds, particularly those found in glycolipids [34].

The poor bioavailability and stability of drugs limits their therapeutic potential, especially when these drugs require access to the central nervous system. By increasing stability in human liver microsomes via the ether bond, UPEI-104 is capable of bypassing first pass metabolism in the liver when injected systemically in an animal. The key advantage to this is that UPEI-104 will be able to remain in circulation longer, increasing its chances of crossing the blood brain barrier. Low metabolic stability and high clearance rates by the liver are the two main mechanisms for losing therapeutic effects of systemically administered compounds [35].

To some extent, by combining LA and apocynin, either UPEI-100 or UPEI-104 may result in neuroprotection via two mechanisms, both as an antioxidant to clean up ROS, and as an inhibitor of NADPH activity. It was previously shown the ester-linked lipoic acid-apocynin conjugate (UPEI-100) inhibited NADPH oxidase and that the effect was more significant than that observed with apocynin alone [27,36].

A limitation of the current study should be mentioned here. From a clinical perspective, the administration of UPEI-104 at 30 min prior to tMCAO was primarily aimed at a potential use (proof of concept) as a preventative therapy (prophylactic use in high risk patients). It would be interesting to determine if UPEI-104 would be neuroprotective when injected following a stroke, however, this would require additional preclinical and subsequent clinically relevant testing to mimic the post-stroke phase in order to suggest its use as a treatment for ischemic cell death (*i.e*., stroke).

## 5. Conclusions

In conclusion, it appears that ether linked compounds are more stable in biological fluid and do not result in a compromise of biological activity. Although the magnitude of the neuroprotective effect of UPEI-104 was similar to that previously described for UPEI-100, it is possible that by bonding apocynin and lipoic acid via an ether bond and increasing the half-life in human liver microsomes, that the therapeutic effect may last longer (beyond the 6 h experimental time course). Subsequent studies will need to be done to determine how much longer past the 6 h window investigated in the current study UPEI-104 could still maintain therapeutic efficacy against ischemia-reperfusion-induced cell death.
